# Investigating the Sociodemographic and Health Characteristics of Non-Sugar Sweeteners Consumption in Greek School-Aged Children: A Cross-Sectional Study

**DOI:** 10.3390/children11070813

**Published:** 2024-07-02

**Authors:** Kyriaki Apergi, Olga Malisova, Antonis Vlassopoulos, Philippa Fidanoglou, Aikaterini Kandyliari, Maria Kapsokefalou

**Affiliations:** 1 Department of Food Science and Technology, University of Patras, 30100 Agrinio, Greece; kyapergi@upatras.gr (K.A.); omalisova@upatras.gr (O.M.);; 2 Food Science and Human Nutrition, Agricultural University of Athens, 11855 Athens, Greece; avlassopoulos@aua.gr (A.V.); kkandyliari@aua.gr (A.K.)

**Keywords:** non-sugar-sweeteners, soft drinks, childhood consumption, parental habits

## Abstract

Background/Objectives: The childhood consumption of non-sugar-sweetened (NSS) soft drinks is a growing concern due to its potential health implications. This study investigated demographic, anthropometric, and lifestyle factors influencing NSS soft drink consumption among children. Methods: A sample of 1304 children and their parents were surveyed. Results: Analysis revealed that nearly 60% reported consuming NSS soft drinks at least once a week. Also, positive associations were found between NSS soft drink consumption and lower socioeconomic status, increased total beverage consumption, higher maternal BMI, and parental soft drink habits. However, upon employing multivariable models, only the association between total and NSS soft drinks consumption remained statistically significant (OR = 18.925, *p* < 0.05 for children; OR = 3.801, *p* < 0.05 for parents), highlighting the pivotal role of parental behavior in shaping children’s consumption patterns. Conclusions: These findings emphasize the importance of tracking parental habits, revealing a strong correlation between parental behavior and children’s soft drink consumption patterns. Understanding these factors is crucial for developing effective public health strategies for children, which should prioritize not only individual behaviors but also parental modeling and household dynamics.

## 1. Introduction

Soft drinks, renowned for their sweetness, effervescence, and flavor, often captivate the palates of children, serving as a prevalent beverage choice that contributes significantly to their daily hydration [1]. However, the consumption of these beverages is associated with a calorie load of approximately 10–20% of the daily energy intake, derived predominantly from added sugars [2]. Thus, excessive intake poses a risk for health outcomes, including weight gain, obesity, as well as gastrointestinal discomfort [3,4].

In 2015, World Health Organization (WHO) recommended free-sugar intake to be less than 10% of total energy intake and, ideally, below 5% [5]. Since then, the substitution of sugar with non-sugar sweeteners (NSS), such as stevia, erythritol, aspartame etc., is a frequently suggested strategy in order to lower sugar consumption. Yet, this target has not been met in many countries, including Greece. In particular, children are a focus group of these efforts, as a high sugar intake poses significant health risks for this age group. Specifically, a notable percentage of children (18.7%) and adolescents (24.5%) in Greece surpassed the recommended limit, obtaining 10% or more of their total energy intake from added sugars, with sweets, refined cereals, and sugar-sweetened (SS) beverages shown to be the main sources of added sugars in the diets of children and adolescents [6].

Thus, the consumption of NSS soft drinks in children has emerged as a subject of scientific research and regulatory attention [7], and regulatory agencies have embarked on extensive research to establish safe daily intake thresholds for various NSS soft drinks, with a particular emphasis on their potential health impacts, especially among the pediatric population. However, recent guidelines from organizations, such as the WHO, have underscored caution regarding the use of NSS soft drinks for weight management and the prevention of noncommunicable diseases (NCDs), urging vigilance in their consumption [8].

The quantification of NSS exposure has been difficult to achieve [9], but it remains important to identify population groups with higher chances of NSS exposure and to focus our efforts on exposure assessment in these potential at-risk groups [10]. Despite the widespread availability of varied NSS products in the Greek market, including beverages, dairy products, and snacks, there is no previous research, to our knowledge, specifically examining the consumption of NSS soft drinks by children in Greece. Globally, studies that have explored NSS beverage consumption among children mention that the consumption rates range from 25.1% [11] to 53% [12] in school children. Based on the existing literature, there are sociodemographic and other characteristics linked to increased soft drink consumption, with socioeconomic status being the most common one, followed by parental practices [13]. Interestingly, these habits seem to have a strong tracking into adulthood even if the socioeconomic status in adulthood is improved [14].

In this study, we aimed to focus on children of similar socioeconomic status and to study soft drink and NSS soft drink consumption between children at risk of food insecurity versus food-secure children and families [15,16]. In particular, we seek to understand whether children who are enrolled in the school lunches program in Greece have different soft drink and NSS soft drink consumption habits. The main hypothesis for this study is that since, in Greece, children are not offered lunch at school unless they are enrolled in a program to combat food insecurity, enrollment in such programs could significantly impact the eating and drinking habits of these children. Previously, we reported differences in eating habits of children enrolled in the school lunches program versus a socioeconomic-level-matched control [17]. Since soft drinks can be consumed during both snacks and main meals, we assumed that having access to a soft-drink-free lunch could impact soft drink and NSS soft drink consumption and, hence, reduce exposure to NSS in total [18,19,20]. A secondary aim of this study was to elucidate the association between NSS consumption and socioeconomic status, anthropometric measurements, lifestyle choices, and demographic characteristics, both among the children themselves and in association with parental characteristics.

## 2. Materials and Methods

### 2.1. Sample

In 2019, a cross-sectional study was conducted during May–June and September–October to investigate factors related to the school lunch program in Greece. The research methodology that followed was approved by the Agricultural University of Athens Research Committee on Research Ethics and Conduct (28, 10 May 2019) and the Hellenic Ministry of Education, Department of Primary Education (Φ.14/ΦΜ/46270/50452/Δ1, 2 April 2019) as required by the Greek law for any study conducted in the school environment during formal school hours. Details about the program’s design can be found elsewhere [21].

In short, participants were selected from schools across Greece participating in the school lunch program as of September 2019. Schools were chosen based on geographical distribution and the presence of both program-participating and control schools nearby. Clearance to enter schools was obtained from each school’s principal, and participant recruitment occurred in two stages. After obtaining written clearance, researchers conducted screening visits at schools, distributing parent and student questionnaires and explaining the study process. Consent forms were given to parents/legal guardians, requesting their approval for questionnaire completion. Consent forms and parent questionnaires were returned within three days, with students instructed to fill out questionnaires independently.

Participants were enrolled in the fifth and sixth grades (aged 11 to 12 years old) of elementary school. In this study 1345 participants (45.1% boys) from five regions of Greece were recruited successfully (participation rate of 30.1%).

### 2.2. Dietary Assessment

Dietary evaluation utilized a semi-quantitative food frequency questionnaire (FFQ) including 48 food groups typical in the local cuisine, which has been validated for the study’s age group as a self-administered tool ([22], Supplement S1). The FFQ incorporated images depicting indicative portion sizes per food group, aiding in quantifying children’s usual consumption relative to these portions. Specific inquiries addressed intake frequencies of certain foods over the previous month, categorized as ‘daily’, ‘3–6 times per week’, ‘2 times per week’, ‘once a week’, ‘1–2 times per month’, and ‘seldom/never’ for food items including SS soft drinks and ‘yes’, ‘sometimes’, and ‘no’ for NSS soft drinks. Total beverages’ consumption was examined as the sum of all the beverages with added sugar, plus juices. For the quantitative analysis of the FFQ about the intake of energy (kcal), macronutrients, fiber, and sodium we used data from Hellenic Health Foundation [23] and the USDA National Nutrient Database [24]. The percentage of energy (kcal) in the diet that came from total beverages was calculated dividing the energy derived from total beverages by total energy consumption. Over- and under-reporting were assessed using the Goldberg cut-off [25], with exclusion criteria based on energy intake to basal metabolic rate (BMR) ratios < 1.16 and >2.65, calculated via the Schofield equations [26].

### 2.3. Anthropometric Assessment

Anthropometry data were self-reported for at-home completion and measured by researchers in the classroom. Anthropometric measurements, conducted by a trained researcher in schools, employed the researcher-assisted approach and occurred approximately 2 h post-meal and before mid-day snack consumption. Weight was measured using a digital scale (Tanita BC-601; TANITA; Tokyo, Japan) with subjects dressed but shoeless, adjusted by subtracting 1 kg to account for clothing. Only weight values were used and not the impedance values due to a lack of standardization for the hydration and exercise status of the children. Additionally, the analysis of body composition was outside of the scope of this study. Standing height, without shoes, was measured using a portable stadiometer (Leicester height-measure) to the nearest 0.1 cm, aligning the head per the Frankfort plane position. In the at-home arm, anthropometric data were self-reported. Body mass index (BMI) was computed by dividing weight (kg) by standing height squared (m^2^), with obesity, overweight, and underweight determined using sex- and age-appropriate z-scores from WHO growth charts [27].

### 2.4. Assessment of Sedentary Behavior

Sedentary lifestyle was evaluated through sleep duration, study duration, and screen time, separately for weekdays and weekends. Screen time encompassed hours spent in front of screens and recreational use of electronic devices. Sedentary behavior was measured as self-reported values of hours spent in each of the above activities daily plus the habitual sleep and wake-up time of the children.

### 2.5. Parental Data

A questionnaire attached to the consent form gathered information on the socioeconomic and demographic characteristics of parents, including age, current weight and height, years of education, annual family income, employment status, and profession. Additionally, parents reported on children’s nutritional preferences, the frequency of family meals, the frequency of meals eaten outside the home, and details on household cooking responsibilities. Parental obesity and overweight status were determined based on self-reported body weight and height, following standard WHO criteria for obesity (BMI ≥ 30.0 kg/m^2^) and overweight (BMI = 25.0–29.9 kg/m^2^) [28].

### 2.6. Statistics

The primary objective was to investigate the demographic, anthropometric, and lifestyle factors influencing the consumption of NSS soft drinks among children. To ensure the validity of the findings, the sample size (n) was calculated to be equal to 1067, using the standard formula for estimating proportions, n = Z^2^ × p × (1 − p)/E^2^, where Z is the Z-value corresponding to the 95% confidence level (1.96), p is the estimated prevalence (0.60), based on previous studies, that about 60% of children consumed NSS soft drinks, and E is the margin of error (0.03). Considering potential non-responses and incomplete data, we inflated our sample size by approximately 20% to ensure the final sample would remain robust. This adjustment led us to target a sample size of around, at least, 1280 children. 

In continuous variables, normality was assessed using the Kolmogorov–Smirnov test and visual inspection of histograms. Descriptive statistics were presented as mean and standard deviation for normally distributed variables and median and IQR (Q1–Q3) for non-normally distributed. Categorical variables were depicted as absolute and relative frequencies. Group differences were analyzed using Pearson’s chi-squared test, while differences in continuous variables between the two groups were calculated using independent sample t-tests for normally distributed variables and Mann–Whitney U tests for non-normally distributed variables. Logistic regression was employed to investigate association between a dichotomous variable (yes or no) that was created for the consumption of NSS soft drink, which served as the as the binary outcome variable, and the children’s or parents’ characteristics, with the strength and direction of these relationships described using Exp(B) as the odds ratios (OR). In the multivariable logistic regression analysis, we examined the combined effect of the statistic significant variables from the univariable models. To address collinearity in multivariable logistic regression, variance inflation factors (VIF) were examined. Statistical significance was determined at *p* < 0.05. All statistical analyses were conducted using R software (version 2023), with acknowledgments to R Core Team (2023) and RStudio Team (2020) for statistical computations and providing an integrated development environment, respectively.

## 3. Results

The sample of the children who participated in the current study consisted of 1304 children. This size allows for adequate power to detect meaningful associations between NSS soft drink consumption and various demographic and lifestyle factors. Table 1 provides a comprehensive overview of the demographic and health-related characteristics of the children, along with parental characteristics.

Among the children, 44.7% were part of the school lunch group and 55.3% were part of the control group. In terms of sex distribution, boys accounted for 45.08% of the sample. The median age of the children was 11.00 years (Q1:11.00–Q3:12.00), with a mean weight of 42.57 kg (SD:10.21). BMI categories revealed a notable proportion of underweight (11.3%) and obesity (3.2%).

Analysis of NSS soft drink consumption showed that 41.33% of children reported no consumption (Q1), while 20.76% consumed 0–1 portions per week (Q2) and 37.91% consumed more than 1 portion per week (Q3), with a mean consumption of 1.06 portions per week. There was a significant difference in the consumption of NSS soft drinks between groups and according to parents’ consumption of soft drinks.

Moving to parental characteristics, the sample comprised 44.96% males, with fathers having an average age of 45.54 years and mothers of 41.77 years. The median BMI for fathers and mothers was 26.82 kg/m^2^ (24.69–28.37) and 23.57 kg/m^2^ (21.41–26.67), respectively. Among the parents, 115 (39.7%) were consumers of soft drinks.

Table 2 presents the results of univariate logistic regression analysis investigating the association between children’s consumption of NSS soft drinks and various demographic, anthropometric, and lifestyle characteristics.

Among the demographic factors, belonging to the school lunch group showed a significant positive association with NSS soft drink consumption (OR = 1.571, *p* < 0.001), indicating that children in this group were almost 1.6 times more likely to consume NSS soft drinks compared to their control. However, sex (boys) did not show a significant association with NSS soft drink consumption (OR = 1.160, *p* = 0.229).

Regarding anthropometric factors, the age of the child exhibited a significant positive association with NSS soft drink consumption (OR = 1.506, *p* < 0.001). In contrast, the BMI category of the child did not show a significant association with NSS soft drink consumption (*p* = 0.388). Notably, children categorized as having a BMI within the category of “overweight” had approximately 17.3% higher odds of consuming NSS soft drinks compared to children in the reference category (normal), and children with BMI within the category of “obesity” had approximately 78.9% higher odds of consuming NSS soft drinks compared to children in the reference category (normal), yet the association was not significant. Children who consumed SS soft drinks had approximately 10 times higher odds of consuming NSS soft drinks compared to those who did not consume soft drinks. For every 10% increase in the total energy derived from total beverages, there was a 46% increase in the odds of NSS consuming soft drinks.

Among parental characteristics, the BMI of the mother (OR = 1.059, *p* = 0.023) and the parents being consumers of soft drinks (OR = 2.836, *p* < 0.001) showed significant positive associations with children’s NSS soft drink consumption. The size of the household did not show a significant association with NSS soft drink consumption among children (OR = 1.054, *p* = 0.445).

The results of the multivariable logistic regression analysis, taking into account the statistic significant variables from the univariable models, revealed several significant associations that are presented in Table 3. Children enrolled in the school lunch group showed a trend towards a 16.2% decrease in the odds of consuming NSS soft drinks, compared to their counterparts, although the association was not statistically significant at the conventional significance level of 0.05 (*p* > 0.05). Similarly, the effect of age lost significance when controlling for other factors, yet every one-year increase in the age of the child increased the odds of consuming NSS soft drinks by approximately 46.2%. In addition, in the multivariable model, there was no significant association between total energy consumption from beverages and either the mother’s BMI or the father’s education. However, in this model, children who consumed SS soft drinks had approximately 18.9 times higher odds of also consuming NSS soft drinks compared to those who avoided all soft drinks, and this association was statistically significant (*p* < 0.05). Also, children whose parents consumed soft drinks had approximately 3.8 times higher odds of consuming NSS soft drinks compared to those whose parents did not consume soft drinks (*p* < 0.05). Based on the provided collinearity statistics (tolerance > 0.75, VIF < 1.34), multicollinearity is not significant among the predictors in our model. Based on the provided collinearity statistics (tolerance > 0.75, VIF < 1.34), multicollinearity is not significant among the predictors in our model.

## 4. Discussion

Non-sugar sweeteners have gained popularity as additives in various products, aiming to reduce calorie intake and discourage excessive sugar consumption, factors associated with the risk of overweight and obesity. Despite their potential benefits in weight management, particularly in the context of rising childhood obesity rates, recent reports from the World Health Organization (WHO) have raised concerns regarding prolonged NSS consumption among adults [8]. Specifically, as indicated by the World Health Organization’s 2023 guidelines on “Use of non-sugar sweeteners,” more long-term studies are needed to fully understand the impacts of prolonged NSS consumption. This study examined various factors influencing NSS soft drink consumption among children. The results suggest that NSS soft drinks were commonly consumed by Greek children in our sample. While several factors such as socioeconomic status, maternal BMI, and total beverage intake were initially associated with NSS soft drink consumption among children, multivariable analyses revealed the influential role of parental behaviors on children’s NSS soft drink consumption, with the most robust predictor of children’s NSS soft drink intake shown to be parents’ and children’s total soft drink consumption.

In the current study, we observed a prevalent use of non-sugar sweeteners in soft drinks, among a representative sample of Greek children, with 58.5% reporting consumption of NSS beverages. To our knowledge, there has been no study specifically investigating NSS consumption among Greek children. While broader research on dietary habits in Greece suggests variability compared to other countries, the prevalence of NSS consumption among Greek children (58.5%) appears notably higher compared to the reported rates in the USA (25.1% in children) [11]. This finding indicates a potentially higher adoption of NSS-containing beverages among Greek youth and highlights the importance of conducting localized studies to understand the factors influencing NSS beverage consumption among Greek youth and to assess potential health impacts in this population.

The lack of an association between NSS soft drink consumption and childhood overweight or obesity has been a topic of interest in nutritional research, including studies involving vulnerable populations, as the majority of this research has been conducted in high-income countries [29]. Preliminary research has unveiled the complexity surrounding the use of NSS soft drink in children to manage excess body weight or prevent weight gain [11]. A systematic review of observational studies found no significant association between NSS soft drink intake during childhood and fat mass accumulation or waist circumference, and a small yet statistically significant association with increased BMI. However, the existing literature is limited by methodological weaknesses, particularly studies being underpowered to fully investigate this hypothesis, highlighting the need for further research in this area [30]. Furthermore, there is limited understanding of how NSS soft drinks impact children’s appetite regulation, raising concerns among experts about potential alterations in their development [30,31,32]. Our findings contribute to this discourse by suggesting that despite including low-income families with a high consumption of NSS beverages in our study, we observed a trend indicating that higher BMI was associated with increased consumption of NSS beverages [33]. However, this association did not reach statistical significance. This underscores the complexity of dietary and other factors influencing childhood obesity in vulnerable populations. Familial influences, socioeconomic factors, and overall dietary patterns may play significant roles that mitigate the impact of NSS beverage consumption on weight status [33]. Moreover, cultural and regional dietary eating patterns may have an exceptional influence on beverage choices and, as a result, on weight and health outcomes. For instance, in Greece, where traditional diets put emphasis on Mediterranean foods, as such fresh, seasonal fruit, and vegetables, but also on family meals at a regular basis, the impact of NSS beverages on obesity risk may differ compared to regions with different dietary patterns [34]. Understanding these contextual factors is crucial for developing targeted interventions to promote healthier beverage choices among vulnerable populations without exacerbating socioeconomic disparities in nutrition access.

Additionally, while some children may consume NSS products without adverse effects, others may experience negative outcomes. To promote healthier dietary choices for children, it is recommended to limit their intake of carbonated beverages and opt for more nutritious alternatives such as water, milk, and 100% fruit juices in moderation [9].

Lower socioeconomic status emerged as a significant predictor of increased NSS soft drink consumption among children. This finding is consistent with the existing literature highlighting the disparities in dietary habits based on socioeconomic factors [20,24,25,26,35,36]. Families facing economic challenges may have limited access to healthier beverage options, such as fresh fruit juices, and may rely more heavily on inexpensive, processed alternatives like NSS soft drinks [37] Addressing socioeconomic inequalities through targeted interventions, such as improving access to affordable healthy foods and beverages in underserved communities, is essential in promoting healthier dietary choices among children [38].

The positive association between maternal BMI and children’s NSS soft drink consumption suggests a potential transmission of dietary habits within families. Mothers often play a primary role in food purchasing, meal preparation, and shaping family dietary habits, thereby exerting a more direct influence on children’s dietary behaviors compared to fathers. As such, maternal eating behaviors and maternal BMI might have a more pronounced impact on children’s food choices, including their consumption of NSS soft drinks [39,40,41]. Additionally, both the quality and quantity of time spent with children may differ between mothers and fathers. Mothers traditionally assume a larger share of caregiving responsibilities, including mealtime interactions, which could further shape children’s dietary habits.

Parental total beverage consumption emerged as a robust predictor of NSS soft drink consumption among children in our study, underscoring the substantial impact of familial influences on children’s dietary behaviors [40,42,43]. Importantly, this relationship remained significant even when controlling for other factors, emphasizing the pivotal role of parental food choices in shaping children’s dietary preferences and habits. In our multivariable model, while several demographic, anthropometric, and lifestyle factors initially showed associations with children’s NSS soft drink intake, the preeminent significance of parental habits may have overshadowed these associations for several reasons [40,43]. Variables such as socioeconomic status or mother’s BMI, which initially appeared significant, may be highly correlated with parental behaviors within the household. Therefore, once parental habits are accounted for in the model, the associations with these variables may become non-significant or attenuated, as their effects may be mediated or explained by parental behavior. Children often learn by observing and imitating the behaviors of their parents, particularly in the context of food and beverage choices [43]. Thus, parental habits may exert a strong and direct influence on children’s dietary preferences and consumption patterns, rendering other factors less influential in predicting NSS soft drink intake among children. Also, parental behaviors may influence children’s access to NSS soft drinks and the availability of them within the household and could shape children’s attitudes and preferences towards these beverages. Consequently, the observed association between parental habits and children’s NSS soft drink consumption may reflect not only parental modeling, but also broader familial and environmental influences that probably could not be fully captured by the other variables included in the multivariable model [41,43,44]. Future research should further explore the mechanisms underlying parental influence on children’s dietary behaviors and develop targeted interventions aimed at promoting healthier family environments and behaviors to mitigate the consumption of NSS soft drinks among children.

## 5. Limitations and Strengths

Our findings should be interpreted in light of certain limitations. The study’s cross-sectional design limits causal inference and the reliance on self-reported data, particularly regarding beverage consumption and lifestyle behaviors (including sedentary behavior), introduces the possibility of recall bias. Despite efforts to control for confounding variables in the multivariable analysis, residual confounding may persist. For instance, we did not account for parental consumption of soft drinks with NSS, which could have provided further insights into familial influences on children’s dietary habits, as these kinds of data were not collected. Additionally, although weight data of children were directly measured (only weight, and not fat-free or fat mass) the same data were self-reported for the parents.

This study contributes to the growing body of literature on childhood dietary behaviors by examining a diverse range of factors influencing NSS soft drink consumption among children. The study employed a large sample size, providing sufficient statistical power to detect associations between variables of interest. Also, the study assessed a wide range of variables, including group enrollment, child’s age, soft drink consumption by both children and parents, the BMI of the mother, and the education of the father. The use of multivariable logistic regression analysis, which allows for the simultaneous examination of multiple predictors while controlling for confounding variables, provides a nuanced understanding of the factors associated with NSS soft drink consumption among children.

## 6. Conclusions

Despite the initial associations observed with various demographic, anthropometric, and lifestyle factors, our multivariable model revealed that the most significant predictor of children’s NSS soft drink intake was the total soft drink consumption by both parents and their children, highlighting the influence of parental habits towards to their children. In conclusion, while certain associations were observed, including the influence of parental and child soft drink consumption on NSS soft drink consumption, the findings underscore the complexity of factors shaping children’s beverage choices.

Due to the lack of proven benefits and the potential health concerns associated with NSS consumption, the WHO has raised concerns regarding the long-term impacts of NSS consumption, advising education in children’s consumption of both NSS and SS beverages, in order to promote healthier dietary habits and overall well-being. Our results align with this perspective and highlight the importance of more comprehensive, long-term research to fully understand the health implications of NSS beverages.

In addition, the findings emphasize the need for comprehensive interventions targeting both school and home environments to promote healthier dietary behaviors among children. While parental influence remains important, school-based programs may play a role in shaping children’s food choices, as schools can integrate comprehensive nutrition education into the curricula to empower children with knowledge about healthy eating habits and the consequences of excessive NSS beverage consumption. In this context, interventions, which aim to reduce consumption of both NSS and SS soft drinks among children, should not only address school meal offerings, but also target parental behaviors and attitudes towards soft drink consumption, by implementing educational initiatives to raise parents’ awareness about the impact of soft drink consumption on children’s health and emphasize the importance of role-modeling healthy behaviors at home. Additionally, it is rather important to strengthen school-based nutrition policies to limit the availability of NSS beverages and promote healthier alternatives like water, milk, and natural fruit juices and launch public health campaigns encouraging families to make healthier beverage choices.

Further research is needed to explore the underlying mechanisms driving these associations, considering factors such as parental modeling, household food environment, and socio-cultural influences. This research is crucial for developing targeted strategies to reduce the consumption of NSS soft drinks among children and promote healthier dietary patterns, aligning with the World Health Organization’s 2023 guidelines, which emphasize the need for further studies on the long-term impacts of non-sugar sweeteners.

## Figures and Tables

**Table 1:** Sample characteristics.

Children’s Characteristics (N = 1304)	
Variable	N (%) or Mean (SD) or Median (IQR: Q1–Q3) *
Group (school lunch group)	595 (44.7%)
Sex (boys)	600 (45.08%)
Age (years) *	11.00 (11.00–12.00)
Weight (kg)	42.57 (10.21)
BMI (kg/m^2^) *	18.89 (16.52–22.16)
BMI categories	
Underweight	151 (11.3%)
Normal	1016 (76.3%)
Overweight	8 (0.6%)
Obesity	43 (3.2%)
Consumption of SS soft drinks (portions per week)	
Seldom or never	326 (24.5%)
1–2 times/month	509 (38.2%0
1–2 times/week	252 (18.9%)
2 times/week	162 (12.2%)
3–6 times/week	82 (6.2%)
Consumption of NSS soft drinks (portions per week)	
No	449 (41.4%)
Sometimes	226 (20.8%)
Yes	409 (37.7%)
Consumption of NSS soft drinks (yes) by sex	
Boys	282 (56.6%)
Girls	353 (60.2%)
Difference (*p*-value)	0.229
Consumption of NSS soft drinks (yes) by group	
School lunch group	318 (64.5%)
Control	317 (53.6%)
Difference (*p*-value)	<0.001
Consumption of NSS soft drinks (yes) by consumption of SS soft drinks	
Consumers of both SS and NSS soft drinks	619 (57.1% of total)
Consumers of SS soft drinks	351 (32.4% of total)
Consumers of NSS soft drinks	16 (1.5% of total)
No consumption of soft drinks	98 (9.0% of total)
Difference (*p*-value)	<0.001
% of total energy from total beverages *	6.36 (3.65–11.63)
Boys	6.41 (2.99–11.95)
Girls	6.30 (3.87–11.54)
Difference (*p*-value)	0.450
School lunch group	6.30 (3.30–10.66)
Control	6.38 (3.21–10.32)
Difference (*p*-value)	0.880
Parents’ characteristics (N = 992)	
Variable	N (%) or Mean (SD) or Median (IQR: Q1–Q3) *
Sex (males)	446 (44.96%)
Age (father) *	45.00 (42.00–48.00)
Age (mother) *	42.00 (39.00–45.00)
BMI (father) *	26.82 (24.69–28.37)
BMI (mother) *	23.57 (21.41–26.67)
Parents’ soft drinks consumption (yes)	115 (39.7%)
Education (years, father) *	12.00 (12.00–15.00)
Education (years, mother) *	14.00 (12.00–16.00)
Size of household *	3.00 (3.00–4.00)

* Median (IQR: Q1–Q3); NSS—Non-sugar sweetened; % of total energy from total beverages include juices, milk, chocolate milk and regular soft drinks; BMI—body mass index, with BMI categories for children calculated based on WHO growth charts; SD—standard deviation. The *p*-value indicates the statistical significance of the difference in soft drink consumption between males and females.

**Table 2:** Results of univariate logistic regression on children’s consumption of NSS soft drinks with demographic, anthropometric, and lifestyle characteristics.

NSS Soft Drinks		
Variable	Unadjusted OR	*p*-Value
Group (school lunch group)	**1.571**	**<0.001**
Sex (boys)	1.160	0.229
Age of child (years)	**1.506**	**<0.001**
Child BMI category		
Obesity	1.789	0.129
Overweight	1.173	0.311
Normal	1.000	
Underweight (reference)	0.746	0.330
% of total energy from total beverages	**1.046**	**0.005**
Children’s consumption of SS soft drinks (yes)	**10.802**	**<0.001**
Age of father (years)	1.014	0.424
Age of mother (years)	1.023	0.254
BMI of father (kg/m^2^)	0.967	0.175
BMI of mother (kg/m^2^)	**1.059**	**0.023**
Education of father (years)	**0.943**	**0.050**
Education of mother (years)	0.959	0.271
Parents’ soft drink consumption (yes)	**2.836**	**<0.001**
Size of household	1.054	0.445

NSS—non-sugar sweetened; SS—sugar sweetened; % of total energy from total beverages include juices, milk, chocolate milk and regular soft drinks; OR: odds ratio; BMI—body mass index, with BMI categories for children calculated based on WHO growth charts; SD—standard deviation. ORs are unadjusted ORs obtained from univariate logistic regression analysis. The *p*-value indicates the statistical significance of the difference in soft drink consumption between males and females.

**Table 3:** Results of multivariable logistic regression on children’s consumption of NSS soft drinks.

Variable	Adjusted OR	*p*-Value	Collinearity Tolerance	VIF
Group (school lunch group)	0.838	0.738	0.882	1.134
Age of child (years)	1.462	0.412	0.923	1.083
% of total energy from total beverages	0.974	0.515	0.855	1.170
Children’s consumption of SS soft drinks (yes)	**18.925**	**0.007**	0.921	1.086
BMI of mother (kg/m^2^)	1.103	0.115	0.924	1.082
Education of father (years)	0.973	0.637	0.879	1.138
Parent’s soft drinks consumption (yes)	**3.801**	**0.015**	0.750	1.333

NSS—Non-sugar sweetened; OR—odds ratio, adjusted for all variables listed in the table.

## Data Availability

The data presented in this study are available on request from the corresponding author. The data are not publicly available due to the confidential nature of some information.

## Supplementary Materials

The following supporting information can be downloaded at: https://www.mdpi.com/article/10.3390/children11070813/s1, Supplement S1. Validation of the food frequency questionnaire.
