# Supplementary material for: Investigating the Sociodemographic and Health Characteristics of Non-Sugar Sweeteners Consumption in Greek School-Aged Children: A Cross-Sectional Study
Source: Children (Basel). 2024 Jul 2;11(7):813. doi: 10.3390/children11070813 (PMC11274966; doi:10.3390/children11070813)
Supplement: Supplementary file 1 [file children-11-00813-s001.zip › children-3060072-supplementary.pdf]

Supplement

Validation of the food frequency questionnaire

Regarding the validation of the applied questionnaires, we utilized a semi-quantitative Food Frequency Questionnaire (FFQ) tailored for our study group. The evaluation of the relative validity and repeatability of the FFQ, was conducted by Farajian P. et al. [1] in a study involving 82 healthy children, aged 11-12 years, recruited from public schools. Each participant completed the FFQ and provided non-consecutive 3-day dietary records, which served as the reference method. The repeatability of the FFQ was assessed by administering it again two weeks later. Analysis using Wilcoxon non-parametric pairwise comparisons and Spearman’s correlation coefficients, adjusted for energy intake, showed no significant differences between the two FFQ administrations for most foods and food groups. This indicated the questionnaire's stability over time and the observed correlations between dietary habits and energy intake suggested that the FFQ is a valid and repeatable tool for investigating dietary habits and nutrient intake among children. Detailed findings, including statistical values and correlations.

1. Farajian, P.; Karasouli, K.; Risvas, G.; Panagiotakos, D.; Zampelas, A. Repeatability and Validity of a Food Frequency and Dietary Habits Questionnaire in Children. *Circulation* **2009**, *119*, e288.
